# Tau Antibody Structure Reveals a Molecular Switch Defining a Pathological Conformation of the Tau Protein

**DOI:** 10.1038/s41598-018-24276-4

**Published:** 2018-04-18

**Authors:** Jessica E. Chukwu, Jan T. Pedersen, Lars Ø. Pedersen, Christiane Volbracht, Einar M. Sigurdsson, Xiang-Peng Kong

**Affiliations:** 1 Departments of Biochemistry & Molecular Pharmacology, New York University School of Medicine, New York, NY USA; 2 Neurodegeneration, H. Lundbeck A/S, DK-2500 Valby, Denmark; 3 Neuroscience & Physiology, & Psychiatry, New York University School of Medicine, New York, NY USA

## Abstract

Tau antibodies have shown therapeutic potential for Alzheimer’s disease and several are in clinical trials. As a microtubule-associated protein, tau relies on dynamic phosphorylation for its normal functions. In tauopathies, it becomes hyperphosphorylated and aggregates into toxic assemblies, which collectively lead to neurodegeneration. Of the phospho-epitopes, the region around Ser396 has received particular attention because of its prominence and stability in tauopathies. Here we report the first structure of a monoclonal tau antibody in complex with the pathologically important phospho-Ser396 residue. Its binding region reveals tau residues Tyr394 to phospho-Ser396 stabilized in a β-strand conformation that is coordinated by a phospho-specific antigen binding site. These details highlight a molecular switch that defines this prominent conformation of tau and ways to target it. Overall, the structure of the antibody-antigen complex clarifies why certain phosphorylation sites in tau are more closely linked to neurodegeneration than others.

## Introduction

Alzheimer’s disease (AD) affects more than 5 million people in the USA alone and is estimated to triple by 2050^1^. Currently, there are no disease-modifying therapies available for AD and related neurodegenerative disorders. Efforts in therapeutic discovery have for some time focused on targeting the amyloid-β (Aβ) peptide, which is produced from cleavage of the amyloid precursor protein (APP), and deposits as plaques that are associated with AD pathology^2^. Whereas the genetics of familial or early-onset AD support the Aβ hypothesis that its aggregation into neurotoxic assemblies causes AD, there has been little success in targeting Aβ in sporadic or late-onset AD, which accounts for 97% of all AD cases^3^. Recent findings have provided further support to the central role of tau in the pathogenesis of AD, and it is now a major diagnostic and therapeutic target for this disease.

Tau is a microtubule-associated protein that supports axonal outgrowth and stability along with transportation of vesicles and organelles in central nervous system (CNS) neurons. Functionally, tau coordinates its microtubule attachment through dynamic phosphorylation, which negatively regulates tau’s ability to promote microtubule assembly^4^. Structurally, tau is a soluble and largely unstructured protein that utilizes its flexibility to remain accessible to kinase and phosphatase attachment^5^. Under pathological conditions, hyperphosphorylated tau (pTau) aggregates into oligomers which can assemble into twisted ribbons, straight filaments, and paired helical filaments (PHFs) and sequentially into neurofibrillary tau tangles (NFTs). Collectively, tau oligomers, larger aggregates, filaments and fibrils are thought to trigger microtubule disassembly, axon degeneration and dendritic spinal collapse^5–7^. These events eventually lead to neuronal death and major cognitive dysfunction. While the cause of AD is still unknown, clinicopathologic studies show collectively a strong correlation between levels of modified tau protein and cognitive impairment^8,9^. In addition, genetic variants of tau are associated with familial frontotemporal dementia, Parkinsonism and other tauopathies^10^. Furthermore, levels of tau in cerebrospinal fluid increase throughout disease progression including pTau at levels 3–4-fold higher than that found in the normal brain^11,12^. These attributes make pTau a target for AD therapeutic development, and antibody therapy is currently the most prominent approach within that field^13^.

It has been shown previously that hyperphosphorylation at amino acids Ser396 and Ser404 (numbered according to the 441 residue 2N4R tau isoform^14^) is a promising target for tau immunotherapy^15–29^. We determined a crystal structure of mAb C5.2, which is a novel antibody that targets the pS396 tau epitope region, in complex with a phosphorylated peptide. Our structure is the first one of a tau Ser396 phosphorylation state-specific antibody (PSSA) and herein we reveal the atomic details of its pTau recognition.

## Methods

### Monoclonal antibody and synthetic peptides

MAb C5.2 was generated by Lundbeck, A/S through mouse immunization^30^. C5.26/BL6 and FVB mice at 2–3 months of age were immunized with Tetanus toxin p30 helper peptide epitope conjugated to phosphorylated tau (386–408)-peptide P30-[TDHGAEIVYK(pS)PVVSGDT(pS)PRHL]^30,31^. The immunogenic peptide was formulated in TiterMax following the vendor’s protocol (Norcross, GA, USA). Mice were injected subcutaneously with 20 µg peptide initially and boosted with 0.5 µg peptide at monthly intervals. Control mice were injected with adjuvant only. The mice were finally boosted without Titermax 3 days prior to fusion of splenocytes with SP-2 cells. Hybridomas were selected for re-cloning cycles after exhibiting positive binding to ELISA plates that had been coated with 1 µg/mL phosphorylated tau 386–408 (pS396/pS404), and having preferential binding to soluble tau (S1) and sarkosyl-insoluble tau (P3) antigens from human AD and rTg4510 brain lysates^32^. Such binding was compared with the binding activity of antibodies to brain lysate from controls, using dot blots and brain lysate coated ELISA or MSD plates, selecting for antibodies that bound to pathological tau and not to normal tau. The tau pathology specific mAb was isotyped and identified as IgG_1_. All animal experiments were performed in accordance with the standard operating procedures of H. Lundbeck A/S, and in accordance with the European Communities Council Directive #86/609, the Danish Executive Orders on Animal Testing No. 88 of 30 January 2013 and No. 253 of 08 March 2013, the directives of the Danish National Committee on Animal Research Ethics, and Danish legislation on experimental animals (license no. 2014-15-0201-00339).

Peptides (Table 1) used in ELISA and crystallization were synthesized by W.M. Keck Biotechnology Resource Center (New Haven, CT) or Genscript Inc. (Paramus, NJ). The lyophilized peptides were solubilized in water to a stock concentration of 10 mg/mL before mixing with the Fab fragment of C5.2 for crystallization.

**Table 1:** Peptides used for ELISA, crystallization and fluorescence polarization experiments.

	ELISA and Crystallization	Fluorescence Polarization
pS396/pS404	^386^TDHGAEIVYKSPVVSGDTSPRHL^408^	FITC-Ahx-^389^GAEIVYKSPVVSGDTSPRHL^408^
pS396	^386^TDHGAEIVYKSPVVSGDTSPRHL^408^	FITC-Ahx-^389^GAEIVYKSPVVSGDTSPRHL^408^
pS404	^386^TDHGAEIVYKSPVVSGDTSPRHL^408^	FITC-Ahx-^389^GAEIVYKSPVVSGDTSPRHL^408^
No phosphorylation	^386^TDHGAEIVYKSPVVSGDTSPRHL^408^	FITC-Ahx-^389^GAEIVYKSPVVSGDTSPRHL^408^

### Animals

rTg4510, tTA and non-Tg littermate F1 mice were bred at Taconic, Denmark. Mice expressing the tTA activator transgenes were maintained on 129S6 background strain (Taconic) and mutant tau responder mice were maintained in the FVB/NCrl background strain (Taconic). Mice were screened by PCR using the following primer pairs 5′-GATTAACAGCGCATTAGAGCTG-3′ and 5′-GCATATGATCAATTCAAGGCCGATAAG-3′ for the tTA activator transgene and 5′-TGAACCAGGATGGCTGAGCC-3′ and 5′-TTGTCATCGCTTCCAGTCCCCG-3′ for the mutant tau responder transgene. rTg4510, tTA and non-Tg littermates were group-housed (5 animals/cage) and received water and food *ad libitum* (Brogaarden, Denmark) as well as environmental enrichment. The light/dark cycle was 12 hours; room temperature was 21 ± 2 °C and a relative humidity of 55% ± 5%. All animal experiments were performed in accordance with the European Communities Council Directive #86/609, the directives of the Danish National Committee on Animal Research Ethics, and Danish legislation on experimental animals (license no. 2014-15-0201-00339).

### Tissue collection

Mice were euthanized by cervical dislocation. Brains were quickly removed, weighed and divided sagittally down the midline to yield two hemispheres. One hemisphere was parted from the cerebellum and hindbrain, the forebrain containing cerebral cortex and hippocampus was snap-frozen on dry ice and stored at −80 °C until use for biochemical analysis. Frontal cortices from 4 AD donors (2 males and 2 females) and 4 non-demented healthy controls (HC, 2 males and 2 females) were obtained from Cambridge Bioscience (Cambridge, UK). The brain tissue samples were fresh-frozen and stored at −80 °C until used for biochemical analysis. All donors were of Caucasian race. Age of AD donors: 76–91 years. Age of the HC donors: 57–80 years. Post-mortem delays: 3–8 hours.

### Tau Biochemistry

Brain tissue extraction was performed as described previously^32^. Tissues were homogenized in 10 volumes of Tris-buffered saline containing protease and phosphatase inhibitors as follows: 50 mM Tris/HCl (pH 7.4); 274 mM NaCl; 5 mM KCl; 1% protease inhibitor mixture (Roche); 1% phosphatase inhibitor cocktail I & II (Sigma); and 1 mM phenylmethylsulfonyl fluoride (PMSF; Sigma). The homogenates were centrifuged at 27,000 × g for 20 min at 4 °C to obtain supernatant (S1) and pellet fractions. Pellets were re-homogenized in 5 volumes of high salt/sucrose buffer (0.8 M NaCl, 10% sucrose, 10 mM Tris/HCl, [pH 7.4], 1 mM EGTA, 1 mM PMSF) and centrifuged as above. The supernatants were collected and incubated with sarkosyl (1% final concentration; Sigma) for one hour at 37 °C, followed by centrifugation at 150,000 × g for one hour at 4 °C to obtain sarkosyl-insoluble pellets, referred to as P3. The P3 pellet was re-suspended in TE buffer (10 mM Tris/HCl [pH 8.0], 1 mM EDTA) to a volume equivalent to half of the original volume used for the brain homogenates. S1 and P3 fractions isolated from the individual 4 AD and 4 HC brains were pooled and used as AD pool and HC pool for western and dot blot analysis.

### Fab fragment production and purification

The Fab fragment of C5.2 (Fab C5.2) was prepared by papain digestion. Briefly, C5.2 IgG and papain (Worthington, Lakewood, NJ) were mixed at a 1:15 molar ratio in a buffer (50 mM Tris [pH 6.8] and 100 mM NaCl) containing 20 mM cysteine hydrochloride (Fisher Scientific, Waltham, MA) and 0.1 M EDTA pH 8. The reaction was incubated for 1 hour at 37 °C and was stopped with the addition of 10 mM iodoacetamide (Bio-Rad, Hercules, CA). The Fab fragments were then isolated from the Fc fragments using a HiTrap Protein A affinity column (GE, Boston, MA). Finally, the Fab fragments were further purified using size-exclusion chromatography. The monodispersed peak containing the soluble Fab fragments was collected and concentrated to about 12 mg/mL for crystallization.

### Western blot

Samples were boiled in 1 × LDS loading buffer and 100 mM DTT. A volume corresponding to 0.5 µl S1 and P3 isolated from 24 weeks old rTg4510 and non-transgenic littermate (non-tg) mice or 5 μl S1 and P3 isolated from four pooled Alzheimer’s disease (AD) and healthy control (HC) brains was loaded on a 4–12% Bis-Tris NuPAGE Gel (LifeTech Novex). After electrophoresis, the proteins were blotted over to a Immobilon–FL PVDF membrane (0.45 μm, IPFL10100, Millipore). The membrane was blocked with SEA blocking buffer (Product #37527, Thermo Fisher). Tau and pTau levels were assessed in the samples using 1 μg/mL C5.2 overnight at 4 °C. A secondary fluorophore conjugated IgG antibody was used (IRDye 680 Goat anti-mouse, LICOR biosciences) and the signal was quantified using Odyssey CLx and Image studio software (LI-COR biosciences).

### Dot blot

1 μl sample of S1 and P3 isolated from 24 weeks old rTg4510 and non-transgenic littermate (non-tg) mice or from four pooled Alzheimer’s disease (AD) and healthy control (HC) brains was spotted on nitrocellulose membranes. S1 and P3 isolated from mice was used at a 1:10 dilution. After blocking with a blocking solution containing 5% nonfat milk and 0.1% Triton-X100 in TBS, the membranes were incubated with 1 µg/mL C5.2 overnight at 4 °C. Membranes were washed, and incubated with a peroxidase-conjugated anti-mouse antibody (1:5000; Jackson ImmunoResearch, West Grove, PA). Bound antibodies were detected using an enhanced chemiluminescence system (ECL PLUS kit; Perkin Elmer).

### Immunohistochemistry

Paraffin sections of 4 μm thickness were prepared from blocks of PFA-fixed prefrontal cortex from AD donors and non-demented controls obtained from Tissue Solutions (Glasgow, UK). The sections were deparaffinised, rehydrated and subjected to antigen retrieval by boiling in 10 mM citrate buffer pH 6 for 5 min. Endogenous peroxidase was quenched for 10 min with 1% H_2_O_2_ in PBS. Sections were then incubated 20 min in blocking buffer followed by overnight incubation in a humidified chamber at 4 °C with primary antibody at 0.1 to 1 μg/mL in incubation buffer. Between each incubation step, the sections were washed 3 × 10 min in wash buffer. Subsequently, the sections were incubated at room temperature in the following solutions; 60 min with a biotinylated secondary Ab diluted 1:200 in wash buffer; 60 min in ABC; 20 min in DAB. Finally, the sections were counterstained with hematoxylin and coverslipped.

### Enzyme-linked Immunosorbent Assay (ELISA)

Immulon 4 HBX (extra high-binding) 96-well microtiter plates (Thermo Scientific) were coated with the same peptide used for crystallization diluted to 2 μg/mL in phosphate-buffered saline (PBS) and left overnight at 4 °C (Table 1). Plates were then washed three times in PBS-T (PBS containing 0.05% Tween-20) and blocked in 5% non-fat milk and 3% bovine serum albumin in PBS for 2 hours at room temperature. Plates were subsequently washed 3 times in PBS-T before C5.2 was serially diluted (8 μg/mL–0.05 ng/mL) in PBS and incubated for 1 hour at room temperature. Then, alkaline phosphatase-conjugated goat anti-mouse IgG (Southern Biotech) was diluted 1:2000 in PBS and incubated for 30 minutes at room temperature. Finally, bound IgG was analyzed by addition of p-nitrophenyl phosphate substrate (Thermo Scientific) and measurement at 405 nm on a VersaMax Microplate Reader.

### Fluorescence Polarization

FITC-labeled peptides used in fluorescence polarization experiments were synthesized by Biomatik (Cambridge, ON, Canada). Fab C5.2 affinity was determined using a fluorescence polarization-based direct binding assay with fluorescein isothiocyanate (FITC)-conjugated peptides (Table 1). The FITC-peptide concentration was kept constant at 300 nM while Fab C5.2 was serially diluted from 675 nM to 0.08 nM. Data was collected at 25 °C using excitation and emission wavelengths of 485 nm and 535 nm, respectively. Each experiment was done in a single 96-well plate and calculated in triplicate (buffer: 50 mM Tris, 100 mM NaCl, 0.1% pluronic F-127). The experiments were performed with a DTX 880 Multimode Detector (Beckman Coulter, Jersey City, NJ). Fluorescence data was reduced using multimode detection software and K_D_ values were calculated in GraphPad Prism 7.0 using a nonlinear regression model.

### Crystallization, data collection, and structure determination

Concentrated Fab C5.2 was mixed with the peptide pS396/pS404 at a 1:10 molar ratio. Crystallization conditions were screened and optimized using the vapor diffusion hanging drop method. Well-diffracted crystals were obtained in a solution containing 25.5% polyethylene glycol 4000, 0.17 M ammonium sulfate and 15% glycerol. X-ray diffraction data were collected at 100 K at a wavelength of 0.979 Å at the Stanford Synchrotron Radiation Lightsource (SSRL) beamline 14–1. The data set to 2.09 Å was processed using the XDS software package^33^ and the structure determined by molecular replacement using an initial model with high sequence similarity (Table 2). Multiple steps of refinement were carried out in COOT^34^ and PHENIX^35^. The final structure analysis was carried out in ICM^36^ and figures were generated with Chimera^37^ and PyMOL (http://pymol.org). The final R_work_ and R_free_ of the model are 21.8% and 27.0%, respectively, with 97.25% of the residues in the preferred regions and 2.67% in the allowed regions of the Ramachandran plot.

**Table 2:** Data collection and refinement statistics.

	C5.2
**Data collection**	
Space group	C 1 2 1
**Cell dimensions**	
*a*, *b*, *c* (Å)	161.16, 40.53, 228.06
α, β, γ (°)	90, 104.07, 90
Resolution (Å)	44.3–2.09 (2.17–2.09)*
*R*_merge_	0.069 (0.54)
*I* /σ*I*	12.3 (2.51)
Completeness (%)	99.5 (99.3)
Redundancy	4.2 (4.2)
**Refinement**	
Resolution (Å)	44.3–2.09 (2.17–2.09)
No. reflections	84763 (8330)
*R*_work_/*R*_free_	21.8%/27%
No. atoms	
Protein	10005
Ligand/ion	15
Water	874
***B*** **-factors**	
Protein	36.8
Ligand/ion	45.2
Water	40.8
**R.m.s. deviations**	
Bond lengths (Å)	0.008
Bond angles (°)	1.24

*Values in parentheses are for highest-resolution shell.

### Data Availability

The x-ray coordinates and structure factors have been deposited in the RCSB Protein Data Bank under accession code 6BB4.

## Results

### C5.2 binding to pathological tau protein

**Figure 1:**
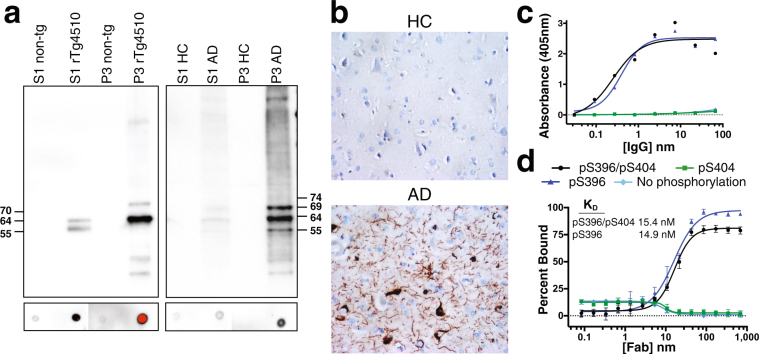
C5.2 specificity for pathological tau protein and high affinity for pSer396. (**a**) Brain samples from 24 weeks old rTg4510 and non-transgenic littermate (non-tg) mice (left image) and from 4 AD and age-matched healthy control (HC) donors (right image) were fractioned into soluble (S1) and sarkosyl-insoluble fraction (P3) and analyzed by western blot (upper image) and dot blot (lower image) for phosphorylated tau at pS396 epitope with C.5.2 (1 µg/ml). AD and HC samples were pooled from 2 males and 2 females each. All four donors were Caucasian and age-matched. Individual AD and HC brains were analyzed with similar results. By western blot, phosphorylated and non-phosphorylated human 4R0N tau with the P301L mutation (tau P301L) from the rTg4510 mice is displayed at 55 kDa, hyperphosphorylated 4R0N tau P301L is mobility-shifted and displayed at 64 and 70 kDa. Phosphorylated and non-phosphorylated six human tau isoforms in AD and HC brains are displayed at 45, 55, 60 and 65 kDa, hyperphosphorylated tau isoforms are displayed at 54, 64, 69 and 74 kDa together with the typical AD smear. (**b**) Representative images of prefrontal cortex from an AD and an age-matched healthy control (HC) donor processed for C5.2 immunohistochemistry. (**c**) C5.2 IgG ELISA and (**d**) C5.2 Fab fluorescence polarization binding curves comparing recognition of four differentially phosphorylated peptides (Table 1). C5.2 binds peptides pS396/pS404 (black) and pS396 (blue), with K_D_ of 15.4 nM and 14.9 nM, respectively, but does not recognize peptide pS404 (green) or the non-phosphorylated (light blue) peptide. The dip in fluorescence for the pS404 and non-phosphorylated peptides around 10 nM Fab concentration is due to protein aggregation and fluorophore quenching. Both experiments confirm C5.2’s phospho-specificity for pS396.

To complement and extend the ELISA/MSD screening for antibody binding to pathological tau fractions (see Methods), we characterized C5.2 binding by Western and dot blots in brain samples isolated from tau transgenic mice (rTg4510 mice overexpressing human tau with P301L mutation) and non-transgenic littermates, as well as from Alzheimer’s disease patients and healthy control subjects. Further characterization was conducted by immunohistochemistry in brain sections from Alzheimer’s disease patients and healthy control subjects. As shown on representative images, C5.2 is specific for pathological tau protein (Fig. 1a,b). It binds predominantly to an insoluble tau fraction (P3) but also has reactivity to a soluble tau fraction (S1) both from rTg4510 mouse and Alzheimer’s disease brains. In the latter, C5.2 recognizes similar bands in both fractions (Fig. 1a). Normal tau in the soluble tau fraction (S1) from non-transgenic littermates and healthy control subjects is not detected with C5.2. On Alzheimer’s disease brain sections, it binds strongly to tangles, pre-tangles and dystrophic neurites, while it does not bind to normal tau in brain sections from healthy control subjects (Fig. 1b).

### C5.2 pSer396 phospho-specificity

In order to evaluate the ability of C5.2 to exclusively target the phosphorylated epitope, we performed an enzyme-linked immunosorbent assay (ELISA). The peptides used in the assay are the same peptides used for crystallization and diluted to a concentration of 2 μg/mL (Table 1). Binding curves were generated from ELISA data by maintaining plate-bound peptide concentrations and serially diluting C5.2 IgG (Fig. 1c). They revealed C5.2’s strong association with pS396/pS404 and pS396 peptides but lack of recognition of pS404 or the non-phosphorylated peptide, suggesting that C5.2 is a pS396 phosphorylation state-specific mAb.

### Nanomolar K_D_ values obtained using fluorescence polarization

To accurately measure the C5.2 binding affinity, we performed fluorescence polarization experiments to determine dissociation constants (K_D_), using the Fab C5.2 and four differentially phosphorylated FITC-conjugated peptides (Table 1). The peptide concentration was kept constant while that of Fab C5.2 was serially diluted from 675 nM to 0.08 nM (Fig. 1d). C5.2 binds peptides pS396/pS404 and pS396 with nanomolar K_D_ values of 15.4 nM and 14.9 nM, respectively, consistent with the ELISA data that C5.2 does not bind the pS404 nor non-phosphorylated peptides. In comparison with a bimodal IgG molecule, each Fab fragment contains a single binding site so the K_D_ was calculated using a nonlinear model.

### Crystal structure of Fab C5.2 in complex with a phosphorylated Tau epitope

**Figure 2:**
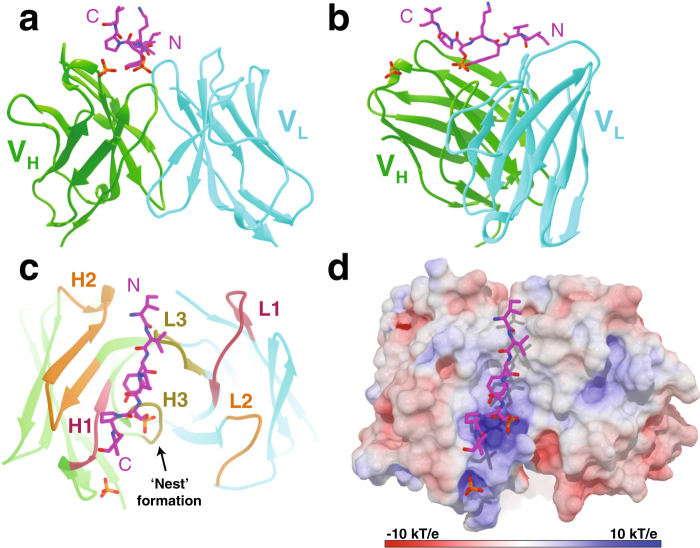
Structure of the Fab C5.2 and pSer396/pSer404 Tau peptide. (**a**) A front view ribbon representation of Fab C5.2 in complex with a pS396/pS404 peptide. Although the peptide used in crystallization is 23 residues in length, only seven residues, containing Ser396 phosphorylation, were visible in the electron density map (^392^IVYKpSPV^398^). The side chain of pSer^396^ and an orphan phosphate are represented as ball and stick. The heavy and light chains are colored light green and cyan, respectively. (**b**) A side view of the Fab C5.2:peptide complex. (**c**) A top view of antigen-binding site annotated by CDR loops. CDR-1, -2, and -3 are colored in salmon, orange and yellow, respectively. (**d**) Molecular surface of the electrostatic potential of the C5.2 antigen-binding site, colored from negative (red) to positive (blue). Note the strong, complementary electropositive surface near the phosphate molecules.

To define the C5.2 epitope, we determined the co-crystal structure of Fab C5.2 in complex with the pS396/pS404 di-phosphorylated tau peptide (^386^TDHGAEIVYKpSPVVSGDTpSPRHL^408^) and refined the structure to a resolution of 2.1 Å with an R_work_ and R_free_ of 21.8% and 27%, respectively (Fig. 2 and Table 2). Although the peptide used in the crystallization is 23 residues in length, only 7 were visible in the electron density map (^P392^IVYKpSPV^P398^). In addition, an orphan phosphate is present near the C-terminal end of the peptide. We attempted to co-crystalize Fab C5.2 with all four peptides from Table 1 but given the low affinity for half of the peptides and the number of crystallization conditions screened, we were only able to resolve one co-crystal structure. The crystals grew in monoclinic space group C2 with three Fab:peptide complexes in the asymmetric unit. The three complexes are highly similar with an average root mean square deviation (RMSD) of 0.2 Å so we only describe one of the complexes hereafter. For clarity, the antibody residues in the text are numbered according to the Kabat convention^38^, with the light and heavy chains labeled with “L” and “H”, but colored cyan and green in the figures, respectively.

### Overall antibody-antigen interactions

**Figure 3:**
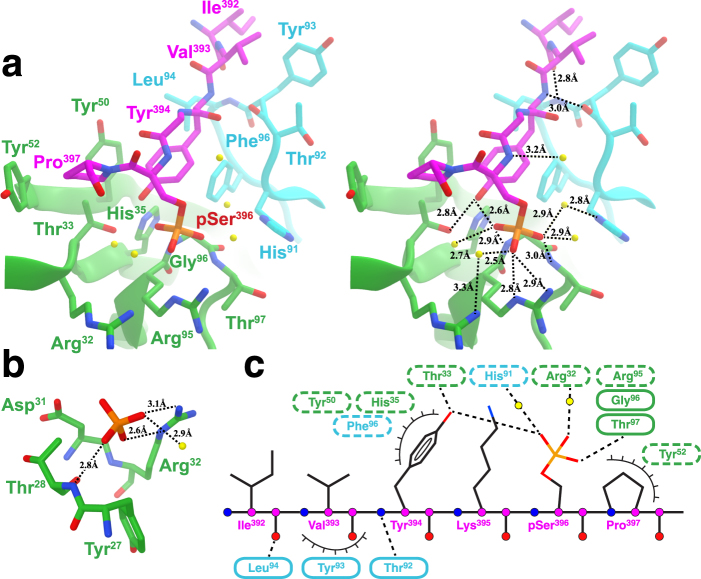
Details of epitope interactions of C5.2. (**a**) A stereo view of key residues involved in antigen binding with their side chains shown as sticks. Note (i) the main-chain hydrogen bonds Thr^L92^ to Tyr^394^ and Leu^L94^ to Ile^392^, (ii) CDR-H3 pSer^396^ phospho-specific contacts from Arg^H32^, Arg^H95^, Gly^H96^, and Thr^H97^ with coordinating water molecules (yellow balls), (iii) intermolecular hydrogen bond between Tyr^394^ and pSer^396^, and (iv) additional contacts from His^L91^ and Thr^H33^. Hydrogen bonds are represented by dashed black lines and labeled with bond distance. (**b**) Key residues from CDR-H1 involved in the orphan phosphate recognition. Note (i) hydrogen bond with Thr^H28^, (ii) salt bridge with Arg^H32^, and (iii) noncovalent contacts with Tyr^H27^ and Asp^H31^. (**c**) A schematic of C5.2 antibody-antigen interactions. Hydrogen bonds are represented by dashed black lines and van der Waals forces denoted by eyelashes. Residues in cyan ovals are from light chain and those in light green ovals from heavy chain. Residues in solid ovals contribute to the interaction by their main chain atoms, and those in dashed ovals contribute to the interaction by their side chain atoms.

C5.2 makes direct contacts with 5 tau residues, including Ile^392^, Val^393^, Tyr^394^, pSer^396^ and Pro^397^. Although Lys^395^ and Val^398^ were also observed in the electron density, they do not form direct contacts with the antibody. The backbone of the epitope region of tau lays straight on top of a relatively shallow (~5 Å deep) antigen-binding groove formed by both heavy chain and light chain CDR regions, with the N-terminus of the epitope located at the heavy chain side and the C-terminus at the light chain side (Fig. 2). The contacts within the antibody-antigen (Ab-Ag) interface involve hydrogen bonds, salt bridges, π-stacking and hydrophobic forces. The negatively charged side chain of pSer396 is located in an electropositive, phosphate-binding pocket coordinated by C5.2 CDR-H residues Arg^H94^, Arg^H95^ and Arg^H32^ (Fig. 2d). These features allow C5.2 to grip the peptide like a claw and facilitate the extended peptide conformation with CDR-H and CDR-L on either side. Although the peptide makes contact with both chains, a majority of them are made with the heavy chain (Fig. 3). We have also observed in the electron density an orphan phosphate close to the C-terminus of the epitope (Fig. 2).

### CDR-L3 coordinates pTau main-chain interactions

CDR-L3 is the only light chain loop that makes contact with the tau peptide. The backbone hydroxyl of Thr^L92^ forms a hydrogen bond with the backbone amine of Tyr^394^. The backbone amine of Leu^L94^ forms a hydrogen bond with the backbone hydroxyl of Ile^392^. In addition, Leu^L94^ and Tyr^L93^ contribute to a hydrophobic environment that facilitates the aliphatic epitope residues Val^393^ and Ile^392^ (Fig. 3a). Lastly, His^L91^ and Phe^L96^, along with heavy chain residues Tyr^H50^ and His^H35^, contribute to a network of aromatic residues in the paratope to provide a stacking interaction with the epitope. The interactions provided by CDR-L3 are main-chain to main-chain and water-mediated hydrogen bonds.

### Tyrosine coordinates a majority of the side-chain interaction

C5.2 contains four tyrosine residues while the epitope contributes a fifth in the antibody-antigen interactions. Tyr^H50^ and Tyr^394^ form a T-shaped stacking interaction that orients the side-chain hydroxyl of Tyr^394^ towards the phosphate of pSer^396^ (Fig. 3a,c). Tyr^394^ of the epitope is then able to form a hydrogen bond with pSer^396^ and Thr^H33^, which further stabilizes the orientation of the epitope. Tyr^H52^ stacks with Pro^397^ and contributes to overall aromatic stacking forces. Tyr^L93^ provides noncovalent contacts and facilitates the aliphatic N-terminal peptide residues. Overall, Tyr^394^, accounting for about 40% of the buried surface area of the epitope, is involved in the most interactions contacting four CDR loops and forming an intramolecular hydrogen bond with pSer^396^ (Fig. 3a,c). Lastly, of interest, is a platform of the aromatic residues Tyr^394^, His^L91^, Phe^L96^, His^H35^ and Tyr^H50^ that stack to suitably mount the peptide for the C5.2 recognition. In addition, Tyr^H27^ is an essential component of the network fostering the orphan phosphate (Fig. 3b). The orphan phosphate ion is a buffer ion and does not suggest additional phospho-peptide interactions with pSer404.

### Phosphorylation-specific antibody interactions

C5.2 is a PSSA that binds the hyperphosphorylated residue Ser^396^. The key feature of pSer^396^ recognition is the turn of the CDR-H3 loop that contains a stretch of residues that coordinate a majority of the phospho-specificity. Arg^H95^ provides a charge-stabilizing salt bridge with a hydroxyl group on pSer^396^ (Fig. 3a). The backbone amine of Thr^H97^ forms a hydrogen bond with another of the hydroxyl group. In between the two residues, Gly^H96^ is tactically located at CDR-H3 hairpin turn to coordinate this nest formation of contacts for pSer^396^ recognition. Gly^H96^ also contributes a hydrogen bond to pSer^396^. While CDR-H3 provides pSer^396^ phospho-specificity, CDR-H1 is responsible for recognition of the orphan phosphate (Fig. 3b). Polar residues Tyr^H27^ and Asp^H31^ provide noncovalent contacts while the backbone amine of Thr^H28^ forms a hydrogen bond with a phosphate hydroxyl. Finally, the side chain of Arg^H32^ forms a salt bridge to complete the nest of interactions orchestrating the orphan phospho-specificity.

### Local conformation of tau peptide

Pathological neurofibrillary tangles and tau proto-filaments are characterized by a high content of β-sheet in a cross-β/β-helix structure^39^. Under normal un-phosphorylated conditions, the tau 393–399 sequence will adopt an α-helical conformation due to the high contents of the β-branched amino-acid valine^40^. Phosphorylation of Ser^396^, like phosphorylation of Ser^199^ with a similar YXpSP motif, can stabilize the secondary structure in a β-strand conformation, forming a seed structure, which may induce tangle filament formation (Supplementary Figs S1 and S2)^41^. In the antibody-tau peptide complex structure, the backbone conformation is locked through the Tyr^394^-pSer^396^ sidechain interaction; the -OH group of Tyr^394^ is only ~2.5 Å away from one of the phosphate oxygens. The dihedral backbone angles (ϕ, ψ) Tyr^394^ (−78.7, 131.4), Lys^395^ (−126.2, 130.1), and pSer^396^ (−56.2, 131.0) are fixed in a β-strand conformation through the strong Tyr^394^-pSer^396^ sidechain interaction. The structure of tau peptide (^392^IVYK^395^) in the C5.2 complex is found to closely resemble (Cα RMSD = 0.2 Å) the core of the tau proto-filament located in the microtubule binding region (MTB) R3 (^306^VQIVYK^311^) that has been recently described as having higher β-strand and amyloidogenic propensity (Supplementary Fig. S2)^42–44^. However, the orientation of the side chain of Tyr^394^ in the C5.2 complex is flipped from that in the β-strand so that Tyr^394^ can interact with pSer^396^. We propose that the YXSP motif in tau constitutes a specific conformational switch which upon serine phosphorylation may change local protein backbone from α-helix to β-strand conformation. This structural phenomenon explains the specificity of this antibody for pathological tau protein.

## Discussion

We have provided the first crystal structure of a mAb targeting the pSer^396^ Tau epitope. The C5.2 Fab:peptide complex structure reveals the peptide in an extended β-strand conformation ranging from tau residues Ile392 to Val398 (^392^IVYKpSPV^398^) and pSer396 serving as a major specificity determinant (Fig. 2). Western blot, immunohistochemistry, ELISA and fluorescence polarization analyses confirm C5.2 to be: (1) specific for pathological tau protein, and; (2) phosphorylation state-specific with nanomolar affinity for the pS396/pS404 and pS396 peptides (K_D_: 15.4 nm and 14.9 nm, respectively) (Fig. 1). Previous studies have described similar pSer396 anti-pTau antibodies^45,46^. The first study characterized the mAbs PHF-1, PHF-13 and PHF-47, which bind di-phosphorylated peptides containing Ser396 phosphorylation. All three mAbs bound pSer396 but did not bind to the non-phosphorylated peptide or to pSer400, pThr403, or pSer404 phosphorylated peptides without the presence of pSer396 phosphorylation^46^. Another study described this pattern of recognition as primary phospho-residue recognition, in this case pSer396, followed by a weak, and possibly non-essential, recognition of a nearby phosphorylated residue^45^. The specificity of pSer396 antibodies for tau pathology was already described in these reports^47,48^, but the structural reason for this specificity has remained unexplained until now.

**Figure 4:**
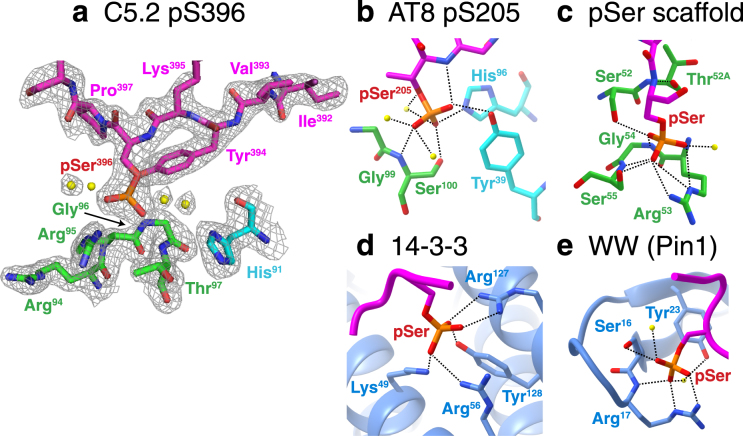
Comparative analysis of anti-pTau mAb and phospho-serine binding motifs. Comparison of phospho-serine binding motifs within (**a**) C5.2, shown with the 2Fo-Fc map at a contour level of 1σ; (**b**) The AT8 anti-tau mAb^51^; (**c**) a pSer/pThr specific scFv scaffold developed using phage display^52^; (**d**) a 14–3–3 domain^60^; and (**e**) the WW domain in Pin1^55^. Figures in panels (**b**–**e**) were generated using structures in the Protein Databank (PDB IDs 5E2W, 4JG0, 1YWT, and 1F8A, respectively). Note the comparable network of hydrogen bonds and salt bridges coordinated by water molecules and the residues Arg, Tyr, Ser, Thr, His, Lys and Gly.

There have been numerous reports of anti-pTau antibodies but only a few have been accompanied by structures at atomic resolution. The structure of the first anti-pTau mAb was published in 2012 and provided a preliminary structural analysis of an avian single chain Fv (scFv), generated using phage display methods^49^, bound to a pT231/pT235 Tau peptide^50^. It gave a comprehensive analysis of phospho-specific antibody recognition but avian antibodies are uncommon and possess a structurally unusual binding pattern. In 2016, the co-crystal structure was described of the AT8 mAb in a complex with a pS202/pT205/pS208 Tau peptide^51^. Although the AT8 epitope contains three phospho-residues, several key residues, including AT8 Gly^H99^, Ser^H100^, Tyr^L39^ and His^L96^, provide the contacts for pThr205 recognition which are similar to the network of C5.2 contacts to pSer396 (Fig. 4a,b).

Our analysis of the antigen binding of C5.2 is an advancement in the field of PSSA that may lead to insights in phospho-serine recognition of PSSA in general. A recent report used phage display to develop PSSAs and designed scFv libraries starting with parent scaffolds of known physiological phospho-residue binding motifs^52^. Their analysis of contacts identified three major regions of recognition, the anchor residue (Ser^H52^ or Thr^H52A^), the conformation stabilizing residue (Gly^H54^) and the more diverse residues conferring side-chain specificity (Arg^H53^, Ser^H55^ and Ser/Thr^H56^). Combining this analysis of pSer specific mAb and that of our C5.2 complex structure, we begin to see a pattern in phospho-serine recognition. In the previous pSer specific scFv, Ser^H52^, Arg^H53^, Gly^H54^ and Ser^H55^ form hydrogen bonds to pSer while Thr^H52A^ anchors the peptide (Fig. 4c). Instead of CDR-H2, C5.2 uses CDR-H3 for phospho-recognition but still generates a similar anion-binding nest for the phospho-serine (Fig. 3).

More interestingly, C5.2 binds the pTau epitope with a similar four-fingered grip as well-known physiological signaling proteins that function through recognition of post-translational phosphorylation. For example, 14–3–3 proteins are a family of dimeric α-helical proteins that recognize two different sequence motifs, R[S/Ar]XpSXP and RX[Ar/S]XpSXP (pS and Ar symbolizing phosphoserine and aromatic, respectively). The key feature of phospho-recognition is a positively charged pocket formed by the residues Lys^49^, Arg^56^, Arg^127^ and Tyr^128^ that provide the ‘four-fingered grip’ (Fig. 4d). The location of proline +2 residues to the C-terminal of pSer plays the important role of kinking the epitope in order to redirect the protein out of the binding interface and allow pSer to extend inward. The WW module is another example of phosphorylation-dependent signaling component that recognizes short proline-rich motifs of PPXY, PPLP and PPR. Intriguingly, the WW domain plays a significant role in tau biochemistry. Pin1, a regulator of cell-cycle progression, contains a WW domain that catalyzes a phosphorylation-dependent prolyl-peptide bond isomerization between pSer/pThr-Pro to render it available for dephosphorylation by the phosphatase PP2A^53,54^. A crystal structure of Pin1 in complex with a di-phosphorylated RNA Pol II CTD hexapeptide (YpSPTpSPS) identifies Ser^16^, Arg^17^ and Tyr^23^ as key contact residues^55^ (Fig. 4e). The four-fingered grip is represented by C5.2 residues Arg^H94^, Arg^H95^, Gly^H96^, and Thr^H97^ (Fig. 4a). Not only do these structures emulate the use of an electropositive binding pocket for phospho-recognition but they give insight into the importance of Pro^397^ flanking pSer396 for epitope recognition and the high frequency of Arg, Tyr, Ser, Thr, His, Lys and Gly residues in the paratope.

The C5.2 structure suggests that the pSer396 phosphate is stabilized by the adjacent Tyr394. The consequence is that the YXpSP becomes a poorer substrate for PP2A. The result will be that phosphorylation at the pSer396 position will accumulate during cellular stress seen in neurodegenerative diseases such as Alzheimer’s disease. Interestingly, Tau pSer199/pSer202 is another phosphorylation site (^196^GYSSPGS^202^) that is frequently associated with PHF pathology in Alzheimer’s disease, which possesses the same sequence motif (YXpSP) as the pSer396 region. This suggests that pSer199 region can also be stabilized in a β-strand (tangle or proto-filament inducing conformation) backbone structure. Solution phase NMR^56^ and solid-state NMR studies of full-length recombinant 2N4R tau^57^ suggested that the preferred secondary structure of the repeat units in the microtubule binding domains (MTB) is β-strand. A recent cryo-EM structure of the MTB domain of tau^39^ suggests that the pathological structure of tangle proto-filaments (tangle monomer) is a cross-β/β-helix unit. The pSer199 and pSer396 YXpSP motifs are flanking both sides of the MTB domains. We thus postulate that the YXpSP motif constitutes a key conformation, which is stabilized in a β-strand through phosphorylation. Specifically, the YXSP → YXpSP phosphorylation reaction amounts to an α-helix to β-strand switch which may change overall backbone conformation of the microtubule domains. Blocking this motif with tau antibodies may have therapeutic benefits as supported by prior work by us and others^15–29,58,59^.

PSSAs are extremely valuable tools for studying dynamic signaling events that require rapid assembly and disassembly of signaling complexes regulated by protein phosphorylation. This includes the microtubule stabilizing function of the tau protein that is disrupted in AD. Using PSSAs to target abnormal phosphorylation has the potential to serve as both a safe therapeutic and a much-needed diagnostic biomarker. These are particularly exciting times for pTau PSSA development with several such antibodies already in clinical trials and additional ones in late stages of preclinical assessment^13^. As more anti-pTau antibodies are developed and characterized, they will provide a framework for optimization of an immunotherapy, as well as a better understanding of AD treatment and prevention.

## Electronic supplementary material

Supplementary Figures
