# Supplementary material for: Tau Antibody Structure Reveals a Molecular Switch Defining a Pathological Conformation of the Tau Protein
Source: Sci Rep. 2018 Apr 18;8:6209. doi: 10.1038/s41598-018-24276-4 (PMC5906480; doi:10.1038/s41598-018-24276-4)
Supplement: Supplementary file 1 — Supplementary Figures [file 41598_2018_24276_MOESM1_ESM.pdf]

Tau Antibody Structure Reveals a Molecular Switch Defining a Pathological Conformation of the Tau Protein

Running Title: Structure of mAb C5.2 bound to pS396 Tau Protein

Jessica E. Chukwu[1], Jan T. Pedersen[2], Lars Ø. Pedersen[2], Christiane Volbracht[2], Einar M. Sigurdsson[3,*], Xiang-Peng Kong[1,*]

Departments of [1]Biochemistry & Molecular Pharmacology, and [3]Neuroscience & Physiology, & Psychiatry, New York University School of Medicine, New York, NY, USA; [2]Neurodegeneration, H. Lundbeck A/S, DK-2500 Valby, Denmark

[*]Correspondence should be addressed to X.P.K. (email: xiangpeng.kong@med.nyu.edu) or E.M.S. (email:einar.sigurdsson@nyumc.org). Requests for mAb C5.2 should be addressed to J.T.P. (JAPT@Lundbeck.com)

Supplementary Figure S1

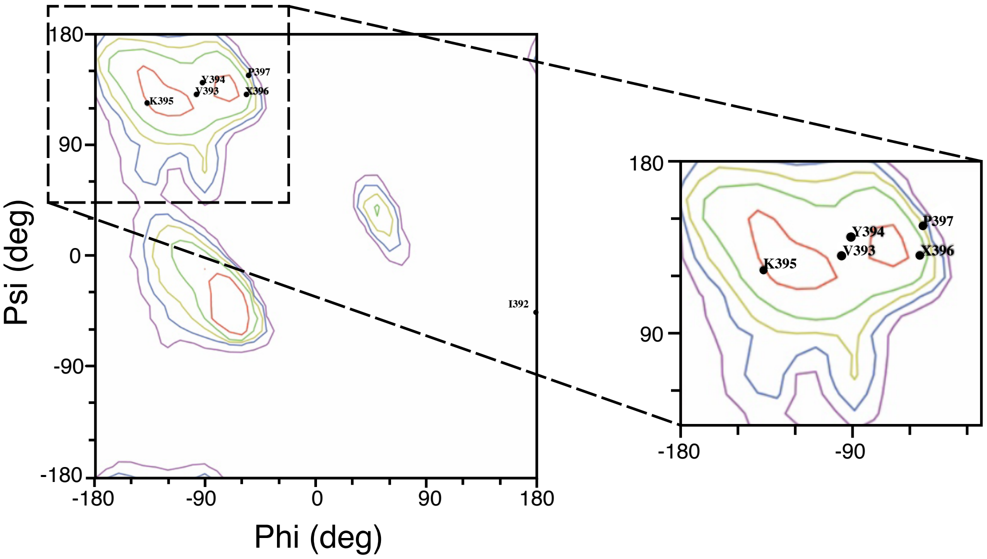

Ramachandran plot showing C5.2 epitope region with dihedral backbone angles in the β-strand conformation. The dihedral backbone angles (ϕ,ψ) Tyr^{394} (-78.7, 131.4), Lys^{395} (-126.2, 130.1), and pSer^{396} (X396; - 56.2, 131.0) are at the upper left-hand corner of the beta-sheet region of the Ramachandran plot.

Supplementary Figure S2

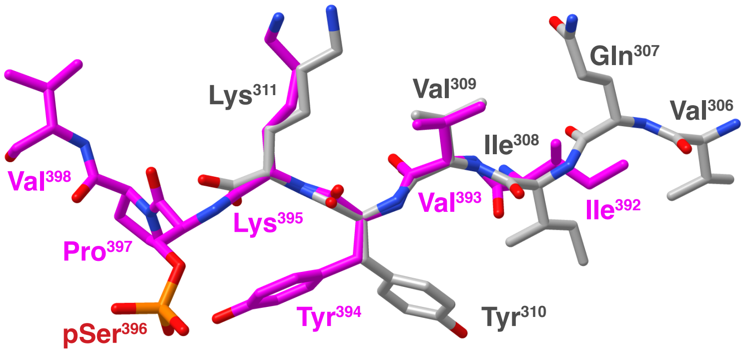

Superposition of C5.2 epitope with tau proto-filament residues 306-311 with the IVYK motif. The structure of tau peptide (^{392}IVYKpSPV^{398}) in the C5.2 complex (magenta) is superimposed (Cα RMSD = 0.2Å) with the core of the tau proto-filament located in the microtubule binding region R3 (^{306}VQIVYK^{311}) that has been recently described as having higher β-strand and amyloidogenic propensity (gray) [38,39]. However, the orientation of the side chain of Tyr^{394} in the C5.2 complex is flipped from that in proto-filament so that Tyr^{394} can interact with pSer^{396}. We propose that the YXS motif in tau constitutes a specific conformational switch which upon phosphorylation may change local protein backbone to a β-strand conformation.
